# The role of Th17 cells in the pathogenesis and treatment of breast cancer

**DOI:** 10.1186/s12935-022-02528-8

**Published:** 2022-03-05

**Authors:** Vahid Karpisheh, Majid Ahmadi, Kazem Abbaszadeh-Goudarzi, Mehran Mohammadpour Saray, Asal Barshidi, Hamed Mohammadi, Mehdi Yousefi, Farhad Jadidi-Niaragh

**Affiliations:** 1 Student Research Committee, Tabriz University of Medical Sciences, Tabriz, Iran; 2 Immunology Research Center, Tabriz University of Medical Sciences, Tabriz, Iran; 3 Stem Cell Research Center, Tabriz University of Medical Sciences, Tabriz, Iran; 4 Cellular and Molecular Research Center, Sabzevar University of Medical Sciences, Sabzevar, Iran; 5 Department of Life Science Engineering, Faculty of New Science and Technologies, University of Tehran, Tehran, Iran; 6 Non-Communicable Diseases Research Center, Alborz University of Medical Sciences, Karaj, Iran; 7 Department of Immunology, Faculty of Medicine, Tabriz University of Medical Sciences, Tabriz, Iran; 8 Research Center for Integrative Medicine in Aging, Aging Research Institute, Tabriz University of Medical Sciences, Tabriz, Iran

**Keywords:** Th17, Breast cancer, Targeted therapy

## Abstract

Breast cancer is a severe problem worldwide due to an increase in mortality and prevalence among women. Despite early diagnostic procedures as well as advanced therapies, more investigation is required to find new treatment targets. Various factors and mechanisms, such as inflammatory conditions, can play a crucial role in cancer progression. Among them, Th17 cells are identified as effective CD4^+^ T cells that play an essential role in autoimmune diseases and inflammation which may be associated with anti-tumor responses. In addition, Th17 cells are one of the main factors involved in cancer, especially breast cancer via the inflammatory process. In tumor immunity, the exact mechanism of Th17 cells is not entirely understood and seems to have a dual function in tumor development. Various studies have reported that cytokines secreted by Th17 cells are in close relation to cancer stem cells and tumor microenvironment. Therefore, they play a critical role in the growth, proliferation, and invasion of tumor cells. On the other hand, most studies have reported that T cells suppress the growth of tumor cells by the induction of immune responses. In patients with breast cancer compared to normal individuals, various studies have been reported that the Th17 population dramatically increases in peripheral blood which results in cancer progression. It seems that Th17 cells by creating inflammatory conditions through the secretion of cytokines, including IL-22, IL-17, TNF-α, IL-21, and IL-6, can significantly enhance breast cancer progression. Therefore, to identify the mechanisms and factors involved in the activation and development of Th17 cells, they can provide an essential role in preventing breast cancer progression. In the present review, the role of Th17 cells in breast cancer progression and its therapeutic potential was investigated.

## Introduction

Although tremendous advances have been made to identify molecular and cellular pathways in carcinogenesis and cancer genetics, cancer is recognized as one of the main reasons for death worldwide. Breast cancer (BC) is one of the deadliest cancer among women. Notably, BC alongside colon and lung cancer, are the three prevalent malignancies worldwide [1]. Various studies globally have shown that 1 out of 8 women has BC, which 90% of all cases are related to lifestyle and environmental factors, while 10% are related to genetic disorders [2]. In developed countries, over the next 25 years, researchers predict that BC incidence and the mortality rate will increase up to 60% [3]. Despite advances in new treatment strategies, such as chemoradiotherapy or chemotherapy combined with surgery, there is a long way to find a practical approach to treat and control BC. Therefore, further studies need to be conducted in order to find new strategies to treat cancer [4].

In tumor microenvironment, accumulated tumor cells can inhibit the immune system's responses through various mechanisms [5]. One of these mechanisms is to develop the inflammatory conditions in the tumor microenvironment, in which tumor cells cause inflammation by infiltration the leukocytes as well as secreting inflammatory cytokines in the tumor site [6–8]. Studies have shown that inflammatory cells and cytokines accumulated in the tumor microenvironment suppress the immune responses and contribute to tumor cells’ growth and development instead of triggering an anti-tumor response [9].

Tumor-associated fibroblasts and tumor cells have been reported to create an inflammatory condition for Th17 cell recruitment [10]. Molecules such as TNF-α, IL-1β, IL-6, IL-21, TGF-β, and IL-23 which are secreted by the immune and tumor cells, accumulated in the TME, can play a vital role in inducing Th17 development [11–13]. Several studies have reported that IL-23 promotes the development of Th17 cells, while TGF-β, IL-6, and retinoid orphan nuclear receptor (ROR) are significant factors in the differentiation of Th17 cells [12, 14–16]. However, some studies have shown that TGF-β can provide an inhibitory function in the differentiation of human Th17 cells [17, 18]. Beside producing and secreting the cytokines such as IL-22, IL-21, and IL-17 [19, 20], Th17 cells have performed an important function in inducing allergic reactions and autoimmune diseases [21]. Numerous studies have reported that Th17 cell populations have been implicated in various cancers, including liver, ovarian, breast, melanoma, and colon [22]. In tumor immunity, the exact mechanism of the Th17 cells is still unknown and seems to have a dual function in tumor development, not only by promoting but also inhibiting. On the one hand, Th17 cells enhance anti-tumor immune responses by stimulating immune cells in the TME, becoming the Th1 phenotype, producing abundant IFN-γ or inducing effector CD8^+^ T cells. In contrast, Th17 cells via secreting IL-17 and exerting immunosuppressive functions, stimulate angiogenesis and inhibit the immune system from growing, respectively, which result in tumor cells development [23]. In BC, the penetration of the Th17 population is a poor prediction factor [24]. In BC patients' peripheral blood, various studies have shown that the population of Th17 cells sharply increases compared to normal individuals, and these cells are more effective than Th1 cells to kill tumor cells [25]. Notably, in BC tissue, Th17 cells are positively related to the IL-6, IL-1β, and IL-17 expression, and also negatively correlated with an increase in the number of metastatic lymph nodes and angiogenesis of tumor cells as well [26]. Therefore, in BC tissue, an increase in Th17 cell population could be an ideal strategy to treat BC in future investigations.

In the present study, we will discuss the Th17 role in tumorigenesis and the treatment of BC.

## Breast cancer and therapeutic strategies

Despite significant advances in cancer treatment, BC is still one of the prevalent health problems. In the coming years, evidence about BC suggests that its incidence and mortality will dramatically increase [27]. Studies have shown that BC is the prevalent malignancy in women under 45-year-old [28]. Due to its complexity and invasive biological characteristics, BC is highly heterogeneous [29]. Although many studies have evaluated BC etiology, little information is available about disease etiology [30]. Numerous environmental and genetic factors significantly increase the risk of BC morbidity and its recurrence. Environmental and lifestyle factors involved in the development of BC include smoking, sedentary lifestyle, obesity, alcoholism, hormone therapy, and ionizing radiation [31–33]. Genetic factors such as family history, ethnicity, and genetic mutations can also increase the risk of BC [34]. Symptoms of BC are different from person to person. In general, BC symptoms include pain, deformity, size changes, abnormal discharge, swelling, redness, dimpling, and lump formation in the breast [35]. There are different strategies to diagnose BC such as mammography, biopsies, blood tests, genomic assays, and imaging tests [36].

Common treatments for BC include hormone therapy, chemotherapy, surgery, molecular targeted therapy, radiation, and immunotherapy [37]. Various factors such as genetics and disease stages were considered to determine the treatment methods. In the early stages of cancer, most patients undergo surgery but often use chemotherapy as the tumor cells progress and metastasize [38]. Endocrine therapies are commonly used for elderly or infirm patients with hormone-positive tumors [39]. BC is currently divided into 4 subtypes: Luminal A (HER2 −/PR +/ER +), Luminal B (HER2 +/PR +/ER + or HER2 −/PR +/ER +), HER2 overexpression (HER2 +/PR −/ER −), and triple-negative BC (TNBC) (HER2 −/PR −/ER −) [40].

Today, with the development of pharmacogenomics and immunology, immunotherapy has become an effective and ideal strategy in BC treatment [41]. In BC, immunotherapy methods such as stimulatory molecule agonist Ab, immune checkpoint therapy, bispecific Ab, and cancer vaccines are among the techniques researchers have focused(Table 1). In BC, scientists hope that new immunotherapy strategies can modify the side effects and current status of common treatments to have effective and better treatment approaches.

**Table 1:** Examples of immunotherapies in clinical trials for breast cancer

Method	Study	Breast Cancer Subtype	Phase	Trial id
Vaccines	CD40L vector vaccine	Breast cancer	I	NCT02140996
Vaccines	HER2 vaccine	Breast cancer	I	NCT01376505
Vaccines	HER2 DC Vaccine	Breast cancer	I/II	NCT02061332
Vaccines	Poly ICLC	TNBC	I	NCT02427581
Vaccines	Trastuzumab + E75	HER2 breast cancer	II	NCT01570036
Vaccines	Chemotherapy + DC vaccine	HER2-/ER + (phase II) and TNBC (phase I)	I/II	NCT02018458
Bispecific antibodies	68Ga-IMP-288 + TF2	CEA + / HER2- breast cancer	I/II	NCT01730612
PD-1	Vinorelbine + Gemcitaine + Eribulin + Capecitabine + Pembrolizumab	TNBC	III	NCT02555657
PD-1	PDR001	TNBC	I/II	NCT02404441
PD-1	Carboplatin + Gemcitabine + Nivolumab + Nab-Paclitaxel	Breast cancer	I	NCT02309177
PD-1	Pembrolizumab	HER2 + breast cancer	I/II	NCT02129556
PD-1	Nivolumab + Ipilimumab + Entinostat	HER2- breast cancer	I	NCT02453620
PD-1	Poly ICLC + CDX-1401 + Pembrolizumab	TNBC	I/II	NCT02661100
PD-L1	Atezolizumab + Entinostat	TNBC	I/II	NCT02708680
PD-L1	Trastuzumab + Durvalumab	HER2 + breast cancer	I	NCT02649686
PD-L1	Atezolizumab	TNBC	II	NCT02478099
PD-L1	Placebo + Nab-Paclitaxel + Atezolizumab	TNBC	III	NCT02425891
PD-L1	Durvalumab + Paclitaxel	TNBC	I/II	NCT02628132
CTLA-4	Ipilimumab + MGA271	TNBC	I	NCT02381314
CTLA-4	Tremelimumab + MEDI4736	HER2- breast cancer	II	NCT02536794
4-1BB	Avelumab + PF-05082566	TNBC	II	NCT02554812
OX40	MEDI6469	Breast cancer	I/II	NCT01862900
LAG-3	Paclitaxel + Placebo + IMP321	Stage IV	II	NCT02614833

## Th17 lymphocytes

Th17 population is a subgroup of CD4^+^ T cells that can produce and secrete various cytokines, including TNF-α, GM-CSF, IL-22, and IL-17 [18]. IL-17-produced by Th17 cells was first identified in the host immune response to *Borrelia burgdorferi* [42]. Vital factors needed for Th17 cell development, including retinoic acid-related (RAR), orphan receptor gamma (ROR-γt), orphan receptor alpha (ROR-α), and STAT3 as a transcription factor. Also, the detection of TGF-β and IL-6 cytokines as Th17 differentiators show that Th17 cells are a separate subgroup of CD4 T cells, easily distinguished from Th1 and Th2 cells [43, 44].

About the site of Th17 cells, the lamina propria is the leading site, but it can be present in epithelial and mucosal tissues when fighting fungi, viruses, and extracellular bacteria [45]. IL-17 produced by Th17 cells can increase the secretion of inflammatory cytokines, including IL-6, TNF-α, and IL-1β, not only by enhancing the production of chemokines such as CXCL-2 and CXCL-8 but also by secreting significant intestinal stimuli such as GM-CSF. In inflammatory tissue, GM-CSF causes the accumulation of granulocytes and granulopoiesis, which lead to inflammation [46–48]. IL-22 and IL-17 cause the secretion of antimicrobial proteins and peptides such as S100 proteins and β-defensins by keratinocytes [49]. Th17 population can play a crucial role in increasing immune responses by enhancing the function of CD8 + T cells and B cells [50–52]. Studies have also shown that there is a direct correlation between Th17 cells and myeloid cells. Ronchi et al*.* have shown that the presence of pertussis toxin (PTX) during immunization causes the production of IL-1β by myeloid cells with CD11b^+^ CCR2^+^ Gr1^+^ phenotype, thus increases the activation of Th17 cells [53]. Also, Pang and colleagues reported that myeloid-derived suppressor cell (MDSC)-derived arginase-1, miR-322-5p, and TGF-β can promote Th17 cell differentiation [54]. Recent studies have reported that Th17 cells are often correlated with various cancers such as lung, breast, prostate, colon, and melanoma [55], and also autoimmune diseases such as multiple sclerosis (MS), inflammatory bowel disease (IBD), and rheumatoid arthritis [56–60].

## Plasticity, differentiation, and development of Th17

**Fig. 1:**
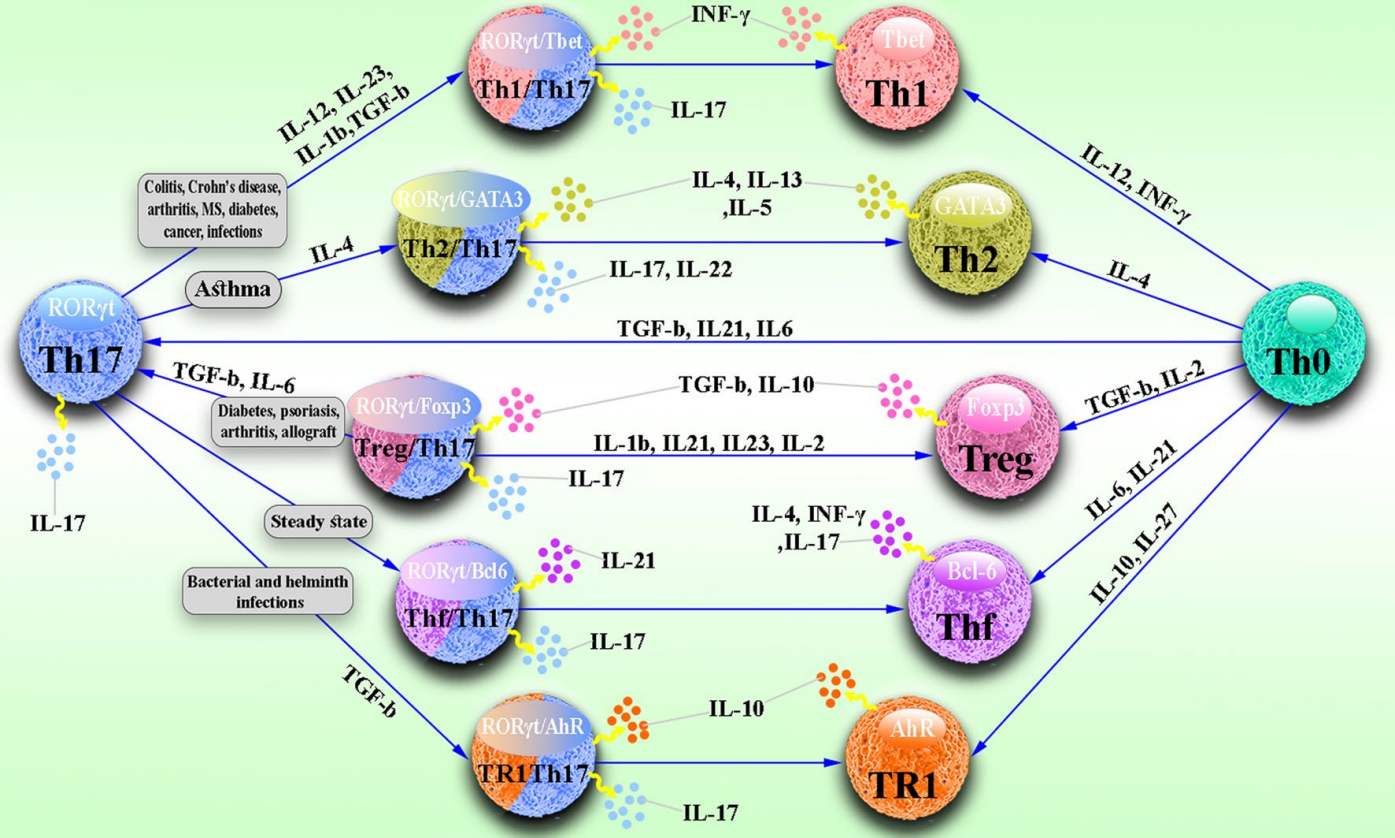
Plasticity of Th17. Th17 cells can mainly transform into TFH, Th2, TR1, Treg, and Th1 cells and exhibit a variety of contrasting functions depending on environmental conditions. During infections and autoimmune diseases, Th17 cells can acquire immune inhibitory functions by becoming TR1 or Treg cells. Th17 cells also change to TFH cells at a steady-state and participate in promoting IgA-producing B cells. Also, Th17 cells acquire pathogenic activity by changing to Th2 cells during asthma or Th1 cells during infection, cancer, and autoimmune diseases

Unlike Th2 and Th1 cells, which are relatively stable, Th17 cells have a very high degree of plasticity [61]. As shown in Fig. 1, studies have shown that Th17 cells can mainly transform into TFH, Th2, TR1, Treg, and Th1 cells which exhibit a variety of contrasting functions depending on environmental conditions. Th17 cells can acquire immune inhibitory functions by changing to TR1 or Treg cells during infections and autoimmune diseases. Th17 cells also modify to TFH cells at a steady state and participate in promoting IgA-producing B cells. Most importantly, Th17 cells acquire pathogenic activity by changing to Th2 cells during asthma or Th1 cells during infection, cancer, and autoimmune diseases [23].

**Fig. 2:**
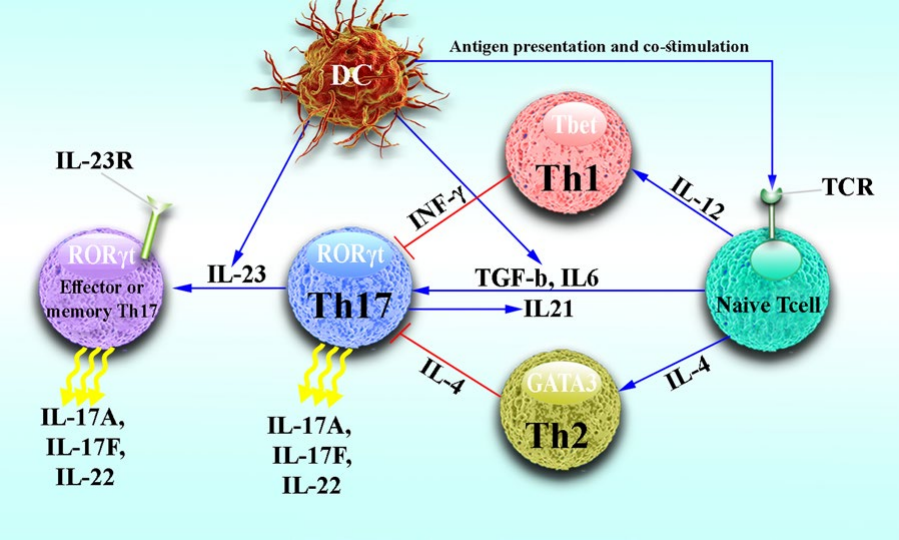
Differentiation of Th17 cell. Naive T cells can be differentiated into three subsets of T helper cells by different cytokines. IL-4 and IL-12 cytokines can increase the differentiation of Th2 and Th1 cells, respectively, and the cytokines secreted by these cells suppress the differentiation of Th17 cells. In contrast, the cytokines IL-23, IL-6, TGF-β, and IL-21 enhance Th17 cell differentiation

For almost two decades, many studies have been conducted on the molecular mechanisms involved in Th17 cell differentiation. Data have shown that various factors and cytokines engaged in this differentiation (Fig. 2) [62]. One of these cytokines is IL-23, which can play an essential role in Th17 cells differentiation by inducing STAT3 as well as inhibiting T-bet and FoxP3 [63]. In naive T cells, it has been confirmed that the expression of IL-23R is in a poor state, that is why it has no role in the early stages of Th17 differentiation from CD4^+^ T cells, but it has a vital role in Th17 activity and final differentiation [43, 64]. However, other key cytokines may be involved in Th17 differentiation, includes TGF-β, IL-21, and IL-6 cytokines. Notably, the TGF-β mechanism in the differentiation of Th17 cells is to suppress transcription factors, including GATA-3 and T-bet, needed for Th2 and Th1 differentiation [65–67]. However, Schumann and colleagues reported that TGF-β could inhibit the production and differentiation of Th17 cells in mice [68]. Despite the auto-proliferative role of IL-21 in Th17 differentiation, the data shows that its absence does not make any difference in Th17 differentiation [69]. Of note, IL-6 can regulate IL-21 function through the STAT3-dependent pathway by direct induction of IL-21 gene expression [70, 71]. Studies have reported that IL-6 can act as a positive enhancer of Th17 differentiation by inducing IL-21 [72, 73]. By signaling pathways such as STAT3, ROR-α, and ROR-γt, Th17 cells are differentiated from other subtypes of T helper cells. Naive T cells produce IL-6 and TGF-β to activate the ROR-α and ROR-γt signaling pathways, which lead to the differentiation of Th17 cells from CD4^+^ T cells [74, 75]. Studies have shown that IL-23, IL-6, and IL-21 cytokines play an essential role in expressing transcription factors which is necessary for Th17 cell differentiation by the activation of STAT3 signaling [63]. In mouse T cells, experiments have shown that mutations or lack of STAT3 expression can result in cancer and metabolic disorders in vivo [76, 77]. It was documented that it could be effective to use antibodies such as ustekinumab, canakinumab, ixekizumab, secukinumab, anakinra, and tocilizumab to suppress Th17 cell differentiation [76, 78–81].

Numerous studies have shown that cytokines required for Th1 differentiation, such as IL-12 and IFNγ, can inhibit the growth and development of Th17 cells [82]. In contrast, IFNγ induces T-bet expression, which stimulates the development of cells by binding and activating the IL-23R promoter. It should be noted that T-bet overexpression reduces IL-17 production [83, 84]. STAT4 and STAT1, as important transcription factors for Th1 differentiation, are significant inhibitors of Th17 development because IL-17 production has been reported to be increased in STAT1-deficient T cells [82]. IL-27 produced by DCs and macrophages, like IFN-γ, can inhibit Th17 cell proliferation by STAT1 activation [85]. Studies have shown that IL-4, STAT6, and GATA-3, involved in Th2 differentiation, can inhibit the development of Th17 cells [82, 86]. IL-25, belonging to the IL-17 cytokine family, can inhibit the development of Th17 by an increase in IL-13 expression. Notably, IL-13 can suppress Th17 development by inhibiting IL-23, IL-1, and IL-6 [87]. Based on previous studies, transcription factors such as STAT5 and cytokine IL-2, are essential factors for Treg cells' development, can effectively inhibit Th17 cell growth [88–90]. About another transcription factor called Ets-1, it also negatively regulates Th17 development by increasing IL-2 production. Deficiency or mutation of Ets-1 increases the IL-23R and IL-22 expression level, thereby promoting the development and differentiation of Th17 [91].

## Th17 in breast cancer

This section will evaluate studies on Th17 function and its potential in BC treatment (Table 2).

**Table 2:** Research correlated to the function of Th17 Cell in breast cancer

Cell line	Treatment	Mice/ Human	In vitro/in vivo	Result	References
–	Anti-FoxP3 and anti-IL-17 antibodies	Human	In vivo	Increasing the population of Th17 cells in breast cancer tissues enhances anti-tumor immune responses	[26]
–	Anti-IFNγ, anti-IL-13, anti-IL-17A, anti-CD8, anti-CD4, anti-CD3 and| anti-CD45	Human	In vivo	Th17 is a new prognostic compound biomarker in TNBC patients	[106]
–	–	Human	In vivo	Serum levels of Th17-related cytokines, including IL-17, TGF-β, and IL-6 in the serum of breast cancer patients, were significantly lower than a healthy individual	[92]
MA782 and 4T1	Anti-CD3 and anti-CD28 antibody	BALB/c mice	In vivo/in vitro	IL-17, one of the most critical cytokines in Th17 cells, was highly expressed in breast tumor tissue	[93]
–	Goat polyclonal anti-human IL-17 antibody	Human	In vivo	IL-17-producing cells accumulated in breast cancer tumor tissue, and these cells were a poor prognostic factor	[56]
–	Anti-PD-1, anti-CD25, anti-CD45RO, anti-CTLA-4, anti-CD103, anti-GITR, anti-CD8, anti-CD3, anti-Foxp3, anti-CD4and anti-IL-17A antibody	Human	In vivo	Th17 and Treg cells increased significantly in the breast cancer tissue	[96]
MCF-7	Anti-Ly6C, anti-CD11b, anti-CD11c, anti-CD40, anti-Gr-1, and anti-F4 80	Human/ BALB/c mice	In vivo/in vitro	IL-17 produced in tumor tissue prevented the accumulation of MDSCs in breast cancer tumor tissue by activating STAT3 signaling	[107]
JB6 Cl41, MCF7, MEF and MDA-MB231	MAP3K8 inhibitor, PD98059, anti-ERK1, SP600125, anti-MEK1/2, anti-STAT3, anti-MAP3K8, anti-c-Jun, anti-IL-22R1, anti- JNK1/2, and anti-IL22 antibody	BALB/c mice	In vivo/in vitro	IL-22, by inducing the expression of Pin1 and MAP3K8, increase the rate of angiogenesis, proliferation, and tumorigenesis of tumor cell	[94]
–	IL- 17A, anti-CD4, anti-CD25, anti-CD127, IgG2a, G1b, anti-CD3 and anti-CD28 antibody	Human	In vivo	Gradually, with breast cancer progression, Treg cells’ accumulation increased, and the population of Th17 cells decreased	[95]
–	Anti-CD28, anti-CCR4, anti-CD39, anti-CD4, anti CD45RA, anti-CD25, anti-CXCR3, anti-CD3, anti-IL-17A, anti-ROR- γt, anti-CCR6and anti-Foxp3 antibody	Human	In vivo	The ectonucleotidase-expressing CD25high + Th17 increases dramatically in tumor tissue and exhibits suppressive function inhibiting CD8 and CD4 cell activation	[97]
MDA-MB-231 and MCF-7	Rabbit anti-sheep anti-rabbit IgG, rabbit anti-human pNF-κB, rabbit anti-human pAKT, Rabbit anti-human IL-17 antibody, rabbit anti-human VEGF, anti-human MMP-9, rabbit anti-human Bcl-2, rabbit anti-human CyclinD1, rabbit anti-human EGFR, anti-human CXCR2, human CXCL1, mouse anti-IL-17A, and mouse anti-CD4 antibody	Human	In vivo/in vitro	Th17 cells regulate CXCL1 expression during cancer progression, and CXCL1 by binding to the CXCR2 receptor could promote the NF-κB/AKT pathway activation, thereby causing the progression of breast cancer	[98]
MDA-MB-231	Anti- TNF-α, anti-IL-17A, anti-CD3, anti- IFN-γ, anti-IL-18Rα, anti CD161, anti-MAIT, anti-Vα7.2 TCR, and anti- CD56 and anti-CD45 antibody	Human	In vitro	Breast tumor cells exposed to bacteria selectively activate Th17-polarized MAIT cells from the mammary ducts, thereby increasing breast cancer progression	[99]
–	–	Human	In vivo	A positive relationship between Th17 cells expressing IL-17A and MIF and increasing the expression of both Th17 and MIF increased breast cancer risk	[100]
4T1	PRI-2191 and Calcitriol anti-mouse CD4, anti-CD335, anti-CD25, anti-CD3, anti-CD19, and anti-CD8a antibody	BALB/c mice	In vivo/in vitro	In young mice treated with PRI-2191, unlike older OVX mice, the expression of Th17, ROR-γt, and ROR-α transcription factors, as well as genes encoding vitamin D receptor and osteopontin, were significantly increased	[108]
-	Anti-Ly6G-, anti-CCR6, anti-Vγ1, anti-VEGFR1, anti- CCR2, anti-IL23R, and anti-IL1R1	KEP, Tcrδ −/−	In vivo	The γδ T cells by production of IL-17A induces neutrophils to promote distant metastases and inhibit CD8 + T Cells and	[101]
MCF7, JB6 Cl41	anti-TPL2, anti-IL-17A, anti- DAB, and anti-PCNA	-	In vitro	IL-17A induces AP-1 activity and the growth and proliferation of breast cancer cells by activating TPL2	[102]
EMT6, MA782,4THM	Anti-IL-I7, anti-CD-3, anti-CD28	BALB/c	In vitro/ In vivo	IL-17 cytokine significantly increased tumor cell proliferation	[93]
4T1, MDA-MB231, EMT6, MDA-MB435, Hs578t	Anti- CD24, anti- CD29, anti- CD25, anti- CD4, anti-CD45, anti-GPR56, anti-Scara5, anti-Tgf β r1, anti-Smad4, anti-Smad2/3, anti-Smad5, anti-Smad6, anti-NF-κB	BALB/c	In vitro/ In vivo	Treg cells induced IL-17RB expression in breast cancer cells by secreting TGF-β1	[103]
MDA-MB-435	Anti- CCR3, anti- GAPDH, anti- IkB alpha, anti-VEGF	CD1-nude	In vivo/ In vitro	The IL-17E cytokine has a high ability to induce anti-tumor responses in vitro and in vivo	[104]
–	Anti- IL-25, anti-CD45, anti-CD45R, anti-CD3, anti-CD11b, anti-CD4, anti-CD8, anti-CD49b, anti-γδTCR, anti-MHC class II, anti-F4/80, anti-CD25, anti-Ly6G, anti-ST2 and anti-IL-17RB	MMTV-PyMT mice	In vivo/ In vitro	IL-17E increase the growth, proliferation, and metastasis of tumor cells	[105]

### The role of Th17 in the progression of breast cancer

By collecting blood samples from 55 BC patients (HER2 +/PR +/ER +) and 34 normal individuals, Baharlou et al., observed that serum levels of Th17-related cytokines, including IL-17, TGF-β, and IL-6, were significantly less than healthy individuals using the ELISA method. They reported that a serum decrease in TGF-β, IL-17, and IL-6 cytokines could be prognostic markers and predictors of the clinical stage of BC. It was concluded that a reduction in serum levels of TGF-β, IL-6, and IL-17 indicated the potential suppressive effects of radiation therapy and chemotherapy on Th17 cells and TGF-β-producing tumor cells in the early stages of BC patients. It was suggested that considering the balance between Th17 and Treg cells using antibodies against Th17-related cytokines could be an ideal strategy for immune therapy in BC [92]. In a study on BALB/c mice, Du and colleagues reported that IL-17 as one of the most critical cytokines of Th17 cells was highly expressed in breast tumor tissue. To determine the level of IL-17 expression in breast tumors, the tumor tissue of mice was examined by ELISA assay on day 5 after inoculation with 4T1 and MA782 cancer cells. They observed that IL-17 increased angiogenesis, metastasis, tumor cells proliferation and growth, and BC progression rate by raising microvessel accumulation in the tumor area. In addition, they stated that injection of IL-17 into tumor-bearing mice dramatically increased tumor progression, whereas in vitro exposure to IL-17 did not contribute to the growth of tumor cells. This means that IL-17 indirectly causes tumor cells to proliferate. That is why this cytokine can be targeted in order to inhibit the development of BC [93]. Accordingly, studying the TME of 207 patients with BC (Luminal A, Luminal B, HER2, Triple-negative), Chen and coworkers examined the frequency of IL-17 secreting cells and its association with pathological and clinical features by immunohistochemical assay. Their data showed that the high level of IL-17 secretory cells was directly related to the triple-negative molecular subgroup, ER/PR negative, and high histological grade in BC. They concluded that Th17 cells could become a major pharmacological target by inhibiting the major regulator ROR-γt, a strategy that could be promising because the predominant role of Th17 cells in several autoimmune diseases has been demonstrated. Therefore, they suggested that these cells were a poor prognostic agent in the TME of BC [56]. In another study on tumor samples of 40 patients (age: 43–73) and 4T1 tumor-bearing mice, it is reported that IL-22, another important cytokine produced by Th17 cells, increased the rate of angiogenesis, proliferation, and tumorigenesis by inducing the expression of Pin1 and MAP3K8, which is directly correlated to the BC progression, as investigated by immunohistochemical staining, western blot, and CAM assay. It was suggested that targeting Pin1 and MAP3K8 could be an ideal approach to reduce BC progression [94].

In a study of 32 patients with BC who underwent surgery (stage I and II-IV), Wang et al*.* measured the frequency of Th17 and Treg cells as well as their cytokine levels using flow cytometry and ELISA assay, respectively. They found that Treg and Th17 cells were highly accumulated in the early stage of BC. In BC progression, the population of Treg and Th17 cells was gradually increased and decreased, respectively. In advanced disease, it was stated that reduced Th17 might reflect the cytokine profile in recurrent and chronic inflammation. In contrast, enhanced Th17 levels in early BC may reflect the cytokine profile in acute inflammation. As a result, to identify the involved mechanisms to regulate Th17/Treg cell balance, BC progression could be effectively prevented [95]. Additionally, in another study on the peripheral blood samples and tumor tissue of 39 patients with BC by immunohistochemistry and RT-PCR assays, it is observed that Th17 and Treg cells significantly increased in the BC tissue, promoting the growth and development of tumor cells. They also found that IL-17A is produced by CD4^+^ and CD8^+^ T lymphocytes was positively related to Th17-related molecules such as CCR6, IL-17A, and ROR-γt. In addition, they reported that vascular endothelial growth factors CXCL8, MMP-2, and MMP-9 were highly expressed by IL-17, there by promoting the growth of tumor cells. These data showed that both tumor invasion and increased IL-17A expression were directly related to the enrichment of Treg cells in invasive breast tumors, suggesting that the regulation of the Treg/Th17 axis could help control BC progression [96].

In a cohort study, Thibaudin and colleagues studied tumor tissues and PBMCs of BC patients (Cohort 1:36 patients who underwent surgery, Cohort 2: 145 patients treated by adjuvant therapy and surgery). ELISA was used to assess cytokine secretion, the immunofluorescence assay to detect the CD73 and CD39 marker, and the immunohistochemistry analysis to investigate the IL-17 and CD8. They reported that ectonucleotidase-expressing CD25^high^ Th17 cells dramatically increased in tumor tissue and exhibited suppressive function via inhibition and activation of CD8 and CD4 cells, respectively. The results showed that these cells not only produced Th17-related cytokines but also induced the Foxp3 and ROR-γt genes. They also found that the cytokines TGF-β and IL-6 were responsible for expressing CD39 ectonucleotidase on these cells. They suggested that the accumulation of ectonucleotidase-expressing CD25^high^ Th17 cells in tumor tissue interfered with the immune system’s antitumor responses. Therefore, targeting these cells could be an attractive way to boost the immune system [97].

Additionally, in another study on 46 patients (age: 23–65) with BC (stage I, II, III, and IV) by immunohistochemistry, ELISA, and flow cytometry assays, it has been shown that Th17 cells regulate CXCL1 expression during cancer progression. It is demonstrated that CXCL1 expressed on tumor cells and its binding to the CXCR2 receptor could promote the activation of the NF-κB/AKT pathway, thereby causing metastasis, angiogenesis, growth, and progression of BC. It is recommended that both downregulation of Th17 and blockade of CXCL1 could help to prevent BC progression [98]. In another study, it is reported that there is a direct link among the breast microbiome functions, mammary duct epithelial cells, and MAIT cells which might play a role in BC progression. These findings showed that breast tumor cells exposed to bacteria selectively activate Th17-polarized MAIT cells from the mammary ducts and enhance BC progression. Therefore, it can be identified how these cells promote the progression of BC, and targeting them may be an ideal therapeutic strategy in future studies [99].

Avalos-Navarro and colleagues, by a study on 150 patients with BC (Luminal A, Luminal B, HER2, Triple-negative), assessed Th17 cytokines profile and the macrophage migration inhibitory factor (MIF) by ELISA assay. They reported there is a positive relationship between Th17 cells expressing IL-17A and MIF. They observed that IL-17A and MIF are higher in aggressive molecular subtypes TN, Luminal B, and HER2 compared to less aggressive Luminal A. Their results showed that an increase in the expression of both Th17 and MIF enhanced the risk of BC, indicating that simultaneous blockade of these factors could be an effective strategy to inhibit BC progression [100].

There are studies that have examined the effect of IL-17 on BC cells and the importance of this cytokine and Th17 cells in the metastasis and invasion of BC. Coffelt and colleagues reported that IL-17-producing neutrophils and γδ cells increased the potency of the invasion and metastasis in BC cells.

It was shown that breast tumor cells induced polarization and proliferation of neutrophils, mediated by granulocyte colony-stimulating factor (G-CSF), which led to the suppression of CD8^+^ T cells as well as an increase in metastasis to surrounding organs. Notably, inhibiting the γδ T cell/IL-17/neutrophil axis could be an effective therapeutic approach in metastatic disease [101]. Kim et al*.* also reported that IL-17A induced AP-1 function and TPL2 activation, which resulted in the growth and proliferation of BC cells. They showed that IL-17A increased the accumulation and growth of JB6 Cl41 cells, while the TPL2 kinase inhibitor prevented IL-17A-induced tumorigenesis. Therefore, blocking this pathway could prevent the proliferation and invasion of BC cells [102]. In another study, Du et al*.* examined the role of IL-17 producing cells in BC cells growth and angiogenesis. Although the exposure of 4T1 mouse BC cells to recombinant IL-17 had little effect on cancer cell proliferation in vitro, administration of this cytokine to cancer mice significantly increased tumor size, associated with increased angiogenesis. In the tumor area, these effects were due to IL-17 secretion from lymphocytes and to a less extent from cancer cells [93]. In another study, Huang and colleagues reported that Treg cells induced IL-17RB expression in BC cells by secreting TGF-β1 and activating the Smad2/4/3 signaling pathway in the tumor-draining LNs (TDLNs), thereby increasing proliferation, angiogenesis, and metastasis of cancer cells. They suggested that blocking IL-17RB could be an effective therapeutic target for inhibiting metastasis and cancer progression [103].

Also, it has been reported that the IL-17E cytokine has a high ability to induce anti-tumor responses in vitro and in vivo. The number of eosinophils and serum level of IL-5 was significantly increased in the peripheral blood of tumor-bearing mice treated with IL-17E, which was related to the anti-tumor activity of IL-17E. They suggested that IL-17E in combination with immunotherapy or chemotherapy could be an ideal treatment approach to reduce the progression of BC [104]. In contrast, Jiang and colleagues reported that IL-17E was significantly secreted by macrophages and CD4^+^ T cells in the MMTV-PyMT breast tumor model. It was demonstrated that inhibition of IL-17E decreased macrophages and type 2T cells in the tumor microenvironment as well as causing a reduction in growth, proliferation, and metastasis of tumor cells. They reported that blocking this cytokine could be a valuable treatment for metastatic BC [105]. In these two current studies, because of the differences in the protocols and working conditions, it is necessary to study the role of IL-17E in the treatment or progression of BC in another investigation.

### The role of Th17 in the preventing breast cancer

Through immunohistological staining and flow cytometry assays, Yang and colleagues indicated that the number of Th17 cells was dramatically increased compared to normal subjects by studying 30 patients with BC. Also, it was found that Th17 cell count was positively related to the expression of IL-6, IL-17, and IL-1β cytokines and negatively associated with an increase in the number of metastatic lymph nodes and tumor cell angiogenesis. Notably, an increase in the number of Th17 cells enhanced anti-tumor immune responses in BC tissues. Therefore, it can be an appropriate treatment choice to enhance the population of Th17 cells in cancerous tissue of BC patients [26]. Accordingly, Faucheux and colleagues studied 106 patients with BC (Luminal A-B, TN, and HER2 +) through Luminex and flow cytometry assays. They reported that Th17 is a new prognostic biomarker in TNBC patients. Due to an increase in Th17 population, it has a good prognosis to identify TNBC. Also, their results confirm a direct correlation between increasing the population of Th17 cells and improving the patients' survival. Thus, the accumulation of Th17 cells can effectively induce anti-tumor immune responses, preventing BC progression in BC tissues [106].

By studying 80 patients with BC and MCF-7 tumor-bearing mice through western blot, RT-PCR, flow cytometry, and ELISA assays, Ma and colleagues demonstrated that myeloid-derived suppressor cells (MDSCs) significantly increased in BC tissue. They reported that produced IL-17 prevented the accumulation of MDSCs in BC tumor tissue by activating STAT3 signaling. They suggested that STAT3 signaling can regulate IL-17 expression. Therefore, a combination of p-STAT3 and IL-17 expressing cells could be an effective treatment strategy for BC [107].

Pawlik et al*.* also examined the expression and activity of Th17 cells in young and old BALB/c mice with 4T1 tumors treated with tacalcitol (PRI-2191) and calcitriol. Their results showed that in young mice treated with PRI-2191, unlike older OVX mice, the expression of Th17 and its cytokines, ROR- γt and ROR-α as transcription factors as well as genes encoding vitamin D receptor and osteopontin were significantly increased. In young mice, it was suggested that treatment with PRI-2191 increased osteopontin expression, which could enhance the differentiation of Th17 cells as an ideal method to treat BC [108].

## Conclusion

Due to the increased mortality and global prevalence of BC especially among women, this disease is a global problem. Today with extensive advances in the medical field, no definitive cure has been reported for BC [27]. However, much research has been done to develop effective treatment strategies and identify the mechanisms and factors involved in the progression of BC. Common systematic treatments for BC include immunotherapy, molecular target therapy, endocrine therapy, radiotherapy, chemotherapy, and surgery [109]. Numerous factors and mechanisms have been identified in BC spread, among which Th17 cells can cause the expansion of BC by producing chemokines, cytokines, and inflammatory pathways [23]. Numerous studies have reported that the frequency of Th17 cells is increased in tumor tissue and PBMC in BC patients. The activity of these cells is dramatically enhanced in patients with advanced disease. However, some studies have reported that Th17 cells inhibit BC progression by promoting the immune system [110, 111]. Observing these contradictions can have various reasons. The first point about these contradictions is the very small number of studies that declare the protective role of Th17 cells in BC patients. Some of these few studies are based on animal models, and it is not easy to generalize the results to human studies. Therefore, it is highly recommended that more comprehensive studies should be conducted in this field. Another point that may shed some light on these discrepancies is the within-patient difference in BC phenotypes, evaluated in different investigations. As we know, different phenotypes of BC patients have many differences in terms of immune responses, disease pathogenesis, and response to treatment. Therefore, in future studies, comprehensive comparisons should be made on the frequency and function of Th17 cells in different disease phenotypes. It is possible to compare these cells in different phenotypes of BC accurately. In many investigations, another point has probably been overlooked is the study of Th17 cells expressing Treg factors such as FoxP3. Studies show that Th17 cells are involved in BC progression by inducing cells that express the markers of Th17 and Treg cells (CD25high Th17 cells). Therefore, it is essential to evaluate these cell population in future studies. Another group of studies that can be beneficial is to use BC transgenic mice lacking Th17 cells. Finally, in mouse models of BC, silencing gene expression of key transcription factors in Th17 cell differentiation can answer many questions about the role of Th17 cells in BC. In addition to cancer, Th17 cells can promote autoimmune and inflammatory diseases such as psoriasis and RA [112, 113]. Due to the critical role of Th17 population in progression of various disorders, understanding mechanisms involved in inducing and activating Th17 cells as well as pathways inhibiting Th17 inflammatory cytokines could be the subject of the exciting field for future studies.

## Data Availability

Not applicable.
